# Revertant mosaicism for family mutations is not observed in *BRCA1/2* phenocopies

**DOI:** 10.1371/journal.pone.0171663

**Published:** 2017-02-15

**Authors:** Jacopo Azzollini, Chiara Pesenti, Luca Ferrari, Laura Fontana, Mariarosaria Calvello, Bernard Peissel, Giorgio Portera, Silvia Tabano, Maria Luisa Carcangiu, Paola Riva, Monica Miozzo, Siranoush Manoukian

**Affiliations:** 1 Medical Genetics Unit, Fondazione IRCCS Istituto Nazionale dei Tumori, Milan, Italy; 2 Department of Pathophysiology & Transplantation, Università degli Studi di Milano, Division of Pathology, Fondazione IRCCS Ca’ Granda Ospedale Maggiore Policlinico, Milan, Italy; 3 Department of Medical Biotechnology and Translational Medicine, Università degli Studi di Milano, Milan, Italy; 4 Pathology Unit, Fondazione IRCCS Istituto Nazionale dei Tumori, Milan, Italy; "Centre de Recherche en Neurosciences de Lyon", FRANCE

## Abstract

In *BRCA1/2* families, early-onset breast cancer (BrCa) cases may be also observed among non-carrier relatives. These women are considered phenocopies and raise difficult counselling issues concerning the selection of the index case and the residual risks estimate in negative family members. Few studies investigated the presence of potential genetic susceptibility factors in phenocopies, mainly focussing on BrCa-associated single-nucleotide polymorphisms. We hypothesized that, as for other Mendelian diseases, a revertant somatic mosaicism, resulting from spontaneous correction of a pathogenic mutation, might occur also in BRCA pedigrees. A putative low-level mosaicism in phenocopies, which has never been investigated, might be the causal factor undetected by standard diagnostic testing. We selected 16 non-carriers BrCa-affected from 15 *BRCA1/2* families, and investigated the presence of mosaicism through MALDI-TOF mass spectrometry. The analyses were performed on available tumour samples (7 cases), blood leukocytes, buccal mucosa and urine samples (2 cases) or on blood only (7 cases). In one family (n.8), real-time PCR was also performed to analyse the phenocopy and her healthy parents. On the 16 phenocopies we did not detect the family mutations neither in the tumour, expected to display the highest mutation frequency, nor in the other analysed tissues. In family 8, all the genotyping assays did not detect mosaicism in the phenocopy or her healthy parents, supporting the hypothesis of a *de novo* occurrence of the *BRCA2* mutation identified in the proband. These results suggest that somatic mosaicism is not likely to be a common phenomenon in *BRCA1/2* families. As our families fulfilled high-risk selection criteria, other genetic factors might be responsible for most of these cases and have a significant impact on risk assessment in *BRCA1/2* families. Finally, we found a *de novo BRCA2* mutation, suggesting that, although rare, this event should be taken into account in the evaluation of high-risk families.

## Introduction

Breast cancer (BrCa) in western countries affects about one in ten women, with only 5–10% of cases presenting a strong inherited component. Germline mutations in the high penetrance *BRCA1* and *BRCA2* (*BRCA1/2*) genes, responsible for the Hereditary Breast and Ovarian Cancer syndrome (HBOC), account for 20–30% of these cases [1]. In families with *BRCA1/2* mutations, carriers are known to be at higher risk for BrCa, which is observed at an earlier age compared with sporadic cases. However, BrCa is also observed among non-carrier relatives, with a frequency of about 10–20% in retrospective and up to 6% in prospective studies [2–6].

These women are considered phenocopies and raise difficult counselling issues concerning the first selection of the index case, in particular for those affected with premenopausal (<50 years) BrCa, and the estimate of the potential residual cancer risks in negative members of families with a strong BrCa history.

To date only a few studies have investigated the presence of potential genetic susceptibility factors underlying the occurrence of phenocopies in *BRCA1/2* families, mainly focussing on BrCa-associated single-nucleotide polymorphisms (SNPs) [2, 5].

We otherwise hypothesized that, as for other Mendelian diseases, a spontaneous correction of a pathogenic mutation, leading to somatic mosaicism, might occur also in BRCA pedigrees, thus determining a greater BrCa risk compared with truly negative cases. This phenomenon, named revertant somatic mosaicism, was observed in patients affected with diverse haematological (including Fanconi anaemia) and non-haematological genetic diseases, mainly affecting the skin, and recent studies suggest that it is not a rare event [7–11]. It is characterised by a complete or partial recovery of somatic cells to the *wild type* genotype through different mechanisms, which include intragenic crossover, gene conversion, back mutation or second-site mutation events [11]. As consequence, the revertant somatic mosaicism leads to a somatic mosaicism occurring in multiple cell lineages. Evidences of revertant mosaicism in HBOC have not been reported to date and genetic modifiers, which might modulate the penetrance in high risk families, are regarded as the most likely predisposing factors for the development of breast cancer in a fraction of non-carriers. Nevertheless, mosaicism in non-carrier relatives has never been specifically investigated and a putative tissue-specific contribution of the familial mutation to the development of cancer has not been ruled out in these patients.

It is, in fact, conceivable that the somatic mosaicism could be undetected. The *BRCA1/2* genetic testing is indeed routinely performed only on DNA from one tissue type, mainly peripheral blood leukocytes (PBLs), and in non-carriers BrCa-affected the family mutation could not be identified due to corrective events occurring in blood cells. In addition, the Sanger sequencing approach could not be enough sensitive to detect low-level mosaicisms.

Based on these considerations, the somatic revertant mosaicism could explain the absence of *BRCA1/2* mutations in the BrCa affected, belonging to positive *BRCA 1/2* families. The presence in an individual of the mutation only in a minor subset of cells, not detectable with standard sequencing approach (i.e.: Sanger sequencing), could still represent a cause underlying the disease in such cases. Moreover, even if at low-level, a putative somatic mosaicism would raise important concerns, especially in relation to the residual risk of developing breast and ovarian cancer as well as of transmitting the mutation to the offspring.

In the present study, we addressed for the first time this issue by evaluating the presence of the family mutation, with a mosaicism-sensitive approach, in 16 patients considered phenocopies based on both clinical and genetic assessment.

## Materials and methods

### Ethics statement

All participants provided a signed informed consent for the use of their biological samples and data for research purposes. The study was approved by the ethics committee of Fondazione IRCCS Istituto Nazionale dei Tumori of Milan.

All the individuals signed the institutional consent for the publication of personal data.

### Patients and samples

We included in this study 16 non-carrier women belonging to 15 families with heterozygous *BRCA1/2* mutations (10 *BRCA1* and 5 *BRCA2*) who developed BrCa before the age of 50 (mean age at diagnosis of first BrCa 43,3 years; median 44,5 years). The only exception is patient 4-e, who was included because affected at the age of 58 with two synchronous tumours belonging to the clinical spectrum of BRCA mutations (BrCa and pancreatic cancer). In addition to patient 4-e, three other phenocopies developed more than one type of tumour: patient 1-b developed a yolk sac tumour at age 20 and BrCa at age 45, patient 14-o developed a non-Hodgkin Lymphoma at age 18, a cervical cancer at age 33 and BrCa at age 43, patient 15-p developed BrCa at age 48 and a melanoma at the age of 50. The pedigrees are shown in S1 Fig.

We evaluated medical history of phenocopies and relatives up to the 4th degree. All the families included in the study resulted eligible for the *BRCA1/2* mutation screening, according to the criteria in use at our institution [12].

All the patients were classified as phenocopies type A, consistent with the definition of Smith and colleagues [2], with the exception of patient 2-c, for whom information about the paternal side of the family was lacking. Moreover, all the phenocopies were first-degree relatives of a proven mutation carrier (type A1 phenocopies), with the exception of patient 7-h, 13-n and 15-p, who were second-degree relatives (type A2 phenocopies).

A significant breast or ovarian cancer history could be identified in either the paternal or the maternal side of the family, with the exception of family 8. In fact, both the phenocopy 8-i and her sister, who was found to carry a *BRCA2* mutation, developed premenopausal BrCa and reported no other BrCa or ovarian cancers cases in both sides of the family. In order to investigate a possible *de novo* origin of the identified mutation, blood samples were collected also from both healthy parents.

For the diagnostic purposes, the investigation of the family mutation was performed by direct sequencing on blood DNA in all patients. In addition, in four of them, who developed BrCa before the age of 40 (2-c, 7-h and 13-n) or bilateral BrCa (10-k), the sequence analysis was extended to the whole *BRCA1/2* coding sequence followed by Multiplex Ligation-dependent Probe Amplification (MLPA). The analyses did not reveal any pathogenic variant in the BRCA genes. Breast tumour features, family mutations and type of the analysed biological sample are shown in Table 1.

**Table 1:** Characteristics of the *BRCA1/2* phenocopies analysed in the study.

Family—Phenocopy	*BRCA1/2* family mutation	Cancer (age)	Breast cancer histology	Sample(s) analysed for mosaicism (MALDI-TOF MS)
**1—a**	*BRCA1* c.1380dupA (p.Phe461Ilefs*19)	Breast (46)	invasive ductal (G3, receptor negative, HER2 negative)	breast tumour
**1 –b**		Yolk Sac (20)	invasive ductal (G1, receptor positive, HER2 negative)	breast tumour
		Breast (45)		
**2—c**	*BRCA2* c.3109C>T (p.Gln1037*)	Breast (36)	invasive ductal (G2, receptor positive, HER2 negative)	breast tumour
**3—d**	*BRCA1* c.190T>C (p.Cys64Arg)	Breast (47)	invasive ductal (G1, receptor positive, HER2 negative)	breast tumour
**4—e**	*BRCA1* c.5266dupC (p.Gln1756Profs*74)	Breast (58)	tubular (G1, receptor positive, HER2 negative)	breast tumour
		Pancreas (58)		
**5—f**	*BRCA1* c.1088delA (p.Asn363Ilefs*11)	Breast (46)	invasive ductal (G2, receptor positive, HER2 negative)	breast tumour
**6—g**	*BRCA1* c.2157_2160delAGAA (p.Lys719Asnfs*16)	Breast (40, 50)	invasive ductal/lobular (G2, receptor negative, HER2 positive)	breast tumour
			invasive ductal (G2, receptor positive, HER2 negative)	
**7—h**	*BRCA1* c.190T>C (p.Cys64Arg)	Breast (32, 38)	invasive medullary (G3, receptor negative, HER2 negative)	blood, urothelium, buccal mucosa
			invasive ductal (G2, receptor negative, HER2 negative)	
**8—i**	*BRCA2* c.771_775delTCAAA (p.Asn257Lysfs*17)	Breast (46)	invasive ductal (G3, receptor positive, HER2 negative)	blood, urothelium, buccal mucosa
**9 –j**	*BRCA1* c.144delG (p.Met48Ilefs*2)	Breast (45)	invasive ductal (G1, receptor positive, HER2 negative)	blood
**10—k**	*BRCA2* c.8487+1G>A	Breast (40)	invasive ductal (G2, receptor positive, HER2 negative)	blood
		BBC (40)	invasive ductal (G2, receptor positive, HER2 negative)	
**11—l**	*BRCA2* c.7806-2A>G	Breast (44)	N.D.	blood
**12—m**	*BRCA2* c.8754+4A>G (p.G2919Vfs*4)	Breast (40)	invasive ductal (G2)	blood
**13—n**	*BRCA1* c.981_982delAT (p.Cys328*)	Breast (36)	invasive ductal/lobular (G2, receptor positive)	blood
**14—o**	*BRCA1* c.798_799delTT (p.Ser267Lysfs*19)	NHL (18)	invasive ductal (G2, receptor positive, HER2 negative)	blood
		Cervical (33)		
		Breast (43)		
**15—p**	*BRCA1* c.190T>C (p.Cys64Arg)	Breast (48)	invasive lobular (G2, receptor positive, HER2 negative)	blood
		Melanoma (50)		

Family mutations (HGVS nomenclature), cancer(s) developed, breast cancer features (histology, hormone receptor and clinical HER2 status) and type of biological sample investigated for mosaicism by matrix-assisted laser desorption ionization time-of-flight mass spectrometry (MALDI-TOF MS) are reported for each patient. “HER2 negative” includes immunohistochemistry scores 0, 1+ or 2+ (not amplified by *in situ* hybridization), “HER2 positive” includes immunohistochemistry scores 2+ (amplified by *in situ* hybridization) or 3+. N.D., not documented.

### DNA isolation

Genomic DNA was extracted from PBLs and buccal swabs using the QIAamp DNA Mini Kit (Qiagen, Hilden, Germany) according to manufacturer’s instructions.

Urine samples were centrifuged at 3500 rpm for 10 minutes, genomic DNA was obtained from the pellet using the QIAmp DNA Micro Kit (Qiagen, Hilden, Germany) following the manufacturer’s protocol.

Tumor DNA was isolated from paraffin-embedded tissue fragments using the Biostic FFPE tissue DNA isolation Kit (MO BIO Laboratories, Carlsbad, CA, U.S.A.), following manufacturer’s instructions. The DNAs were quantified by a spectrophotometer and stored at -20°C.

### High sensitive mutation analyses

In order to verify in the 16 phenocopies the presence of possible low-level mosaicisms for the family mutations, we exploited methods more sensitive in mutation detection, compared with Sanger sequencing.

#### MALDI-TOF mass spectrometry

Matrix-assisted laser desorption ionization time-of-flight mass spectrometry (MALDI-TOF MS) is a very sensitive method for the detection of a mutation in a mosaic condition (~5% mutation detection threshold) [13].

Familial *BRCA1/2* mutations were analysed by the MassArray iPLEX platform (Agena Bioscience, Hamburg, Germany), based on a MALDI-TOF mass spectrometry. In order to determine the sensitivity threshold of the method, DNA from c.771_775delTCAAA *BRCA2* mutation carrier (family 8) was subjected to serial dilutions with a control *wild type* DNA (25%, 12.5%, 2.5% and 1.25% of the mutant allele) and subsequently processed. A peak highlighting the mutation was visible even with a proportion of mutant allele as low as 2.5% (mass spectra available upon request).

The PCR and extended primers were manually designed to detect the nucleotide changes. Specific oligonucleotide tag sequences were added to the 5’-end of each PCR primer to optimize the PCR reaction (primers available in S1 Table). The PCR amplification was performed in 5μl reaction mixture containing 30ng of DNA, each designed primers at 100nM concentration, 100nM dNTP mix, PCR buffer, 25mM MgCl2, and 5U Taq DNA polymerase (Agena Bioscience, Hamburg, Germany). The mixture was incubated as follows: 95°C for 2 min, 45 cycles at 95°C for 30 s, 56°C for 30 s, and 72°C for 60 s, and then a final extension step at 72°C for 5 min. The remaining unincorporated dNTPs were dephosphorylated and inactivated by the treatment with 1.7U of Shrimp Alkaline Phosphatase (SAP). The plates were incubated at 37°C for 40 min and then at 85°C for 5 min.

The iPLEX reaction mix, containing iPLEX Buffer Plus, iPLEX Termination Mix, iPLEX enzyme (Agena Bioscience, Hamburg, Germany) and extended primers, was added to the PCR amplification products. The iPLEX reaction was carried out in the following conditions: 94°C for 30 s, 40 cycles at 94°C for 5 s [52°C for 5 s and 80°C for 5 s (repeated five times per cycle)] and a final extension step at 72°C for 3 min. The samples were spotted on a SpectroCHIP (Agena Bioscience, Hamburg, Germany), which was analysed by mass spectrometry. The spectrum profiles generated by MALDI-TOF MS were acquired and examined with the SpectroTYPER 4.0 software (Sequenom, Inc., San Diego, CA).

Based on the availability of the tissues, the analysis was performed on breast tumour DNA in 7 out of 16 patients (1-a, 1-b, 2-c, 3-d, 4-e, 5-f and 6-g) and on blood, buccal mucosa and urine samples in 2 other patients (7-h, 8-i). In the remaining 7 cases, the analysis was carried out on PBLs DNA.

In family 8, the analysis was carried out also on blood DNA of the patient’s parents. From each family, PBLs DNA of mutation carriers were used in each assay as positive controls. *Wild-type* DNA was analysed with each assay as a negative control.

#### TaqMan SNP genotyping assay and STRs segregation analysis

TaqMan assays for SNP genotyping by real-time PCR was used as an additional, very sensitive method to analyse the family 8, in order to verify at first the presence of a low-level mosaicism of the *BRCA2* mutation c.771_775delTCAAA in PBLs and buccal mucosa cells of the phenocopy 8-i. Subsequently, the same assay was used on both parent’s PBLs DNA, in order to detect a possible low-level mosaicism in either of the two. The non-paternity, that could explain the absence in both parents of the mutation present in one daughter, was excluded by a short tandem repeats (STRs) segregation analysis using the forensic kit PowerPlex® ESX 17 System of Promega, which allows the four-color fluorescent detection of 17 loci (D18S51, D21S11, TH01, D3S1358, Amelogenin, D16S539, D2S1338, D1S1656, D10S1248, FGA, D8S1179, vWA, D22S1045, SE33, D19S433, D12S391 and D2S441).

The genotyping of the *BRCA2* mutation was performed using TaqMan® SNP genotyping assay on the Applied Biosystems StepOne™ real-time polymerase chain reaction (PCR) system. TaqMan Universal Master Mix (catalog number: 4440045) and Custom TaqMan SNP genotyping assay (catalog number: 4332077) were obtained from ThermoFisher Scientific (Paisley, UK). Each reaction was 25 μL consisting of 11.25 μL of 20 ng of genomic DNA, 12.5 μL of 2X TaqMan Universal Master Mix, and 1.25 μL of 20X TaqMan SNP genotyping assay (diluted by 1X TE buffer, pH = 8). PCR cycling conditions were as follows: pre PCR read 60°C for 30s, 95°C for 10 min; followed by 50 cycles of 92°C for 15 s, 60°C for 1min and a post PCR read at 60°C for 30s. The fluorescence intensity in the VIC and FAM channels was measured at the end of each cycle. Results were analysed by StepOne software (Applied Biosystems, Grand Island, USA). The genotypes for the *BRCA2* mutation c.771_775delTCAAA were evaluated in the allele discrimination plot, that establishes the genotype of every sample on the basis of the fluorescence signal intensity of the FAM and VIC dyes. Serial dilutions of the *BRCA2* c.771_775delTCAAA mutation carrier with a control *wild type* DNA were obtained (25%, 12.5%, 2.5%, 1.25% and 0.5% of the mutant allele) and analysed as above. A signal corresponding to the mutant allele was detected at a proportion as low as 1.25% (data available upon request). The experiments were repeated at least 3 times with consistent results. Primer and probe sequences are available in S1 Table.

## Results

The MALDI TOF-MS analysis (~5% mutation detection threshold) was performed on the 16 phenocopies.

In 7 cases (from families 1–6) we were able to retrieve for the analysis breast tumour samples, in which a putative mosaicism for a *BRCA1/2* mutation should be detected at a higher frequency compared with other tissues. In patients 7-h and 8-i the analysis was performed on PBLs, buccal mucosa and urinary tract cells. The other 7 patients (from families 9–15), for whom additional tissues were not available, MALDI TOF-MS was carried out on PBLs DNA only. In all the investigated phenocopies, the respective family mutations were not detected in any of the analysed tissues.

**Fig 1:**
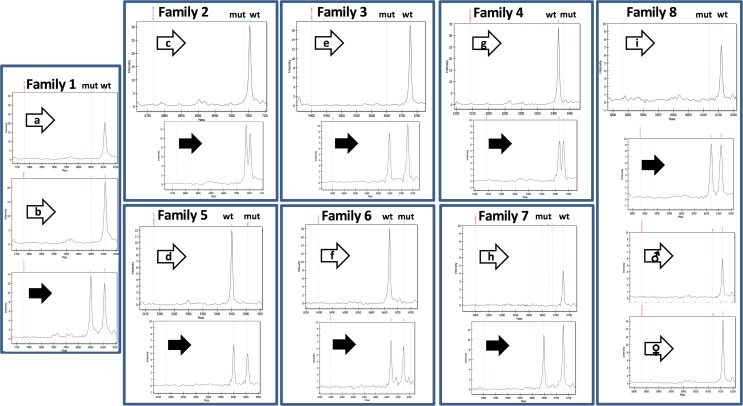
Mass spectra of phenocopies (white arrows) and mutation carriers (black arrows) from families 1–8. Mutant (mut) and *wild type* (wt) alleles are discriminated by the difference in size. Mass spectra of patients a-g (Families 1–7) refer to DNA extracted from breast tumour; for patients h-i (Families 7–8) the results shown refer to the analysis on DNA extracted from urine samples. The analysis of the father (♂) and mother (♀) of patient 8-i was carried out on blood DNA.

Fig 1 shows the mass spectra of families 1–8, in which the analysis was performed on tumour samples or other ecto/endodermal tissues of the affected phenocopies, and on PBLs DNA of heterozygous *BRCA1/2* mutation carriers from each family. Mass spectra of the other seven patients from families 9–15, for whom the analysis was carried out on PBLs DNA only, are shown in S2 Fig.

**Fig 2:**
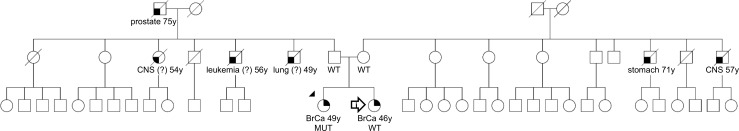
Pedigree of family 8. The white arrow indicates the phenocopy 8-i; the black arrowhead indicates the family proband (i.e. the first tested family member), carrier of the *de novo BRCA2* mutation c.771_775delTCAAA (p.Asn257Lysfs*17). Genetic analysis results (MUT, mutation carrier; WT, *wild-type*) are reported beneath tested individuals; neoplastic diseases and age at diagnosis in years (y) are reported beneath affected individuals. BrCa, breast cancer; CNS, central nervous system;?, uncertain data.

Additional studies were performed in family 8, due to the presence of healthy parents and, with the exception of the proband and her sister, no other breast and ovarian cancer cases in both sides of the family (Fig 2). The MALDI-TOF-MS analysis of the phenocopy (8-i) excluded the presence of the family mutation in PBLs, buccal mucosa and urinary tract cells. The same analysis, extended to PBLs DNA of the parents, showed that the mutation was not present in either of the two.

In order to investigate a non-paternity, we performed a STRs segregation analysis of 17 loci in both affected sisters and their parents. The analysis confirmed the presence of the paternal alleles in both the proband and the phenocopy, thus excluding a non-paternity (data available upon request).

In order to further investigate the presence of somatic mosaicism in patient 8-i using an additional more sensitive technique, we performed a quantitative genotyping assay by real-time PCR (~1% mutation detection threshold) of the *BRCA2* c.771_775delTCAAA mutation, which was not detected in PBLs and buccal mucosa cells of the phenocopy. A putative somatic mosaicism in the patient’s parents was also investigated through the real-time PCR approach, and neither parent resulted to carry the mutation even at a low frequency. These results, on the whole, support the hypothesis of a *de novo* occurrence of the mutation identified in the family proband.

## Discussion

Whether or not individuals testing negative, from BRCA families, are at increased risk for BrCa is still debated. A first retrospective study [2] of selected high-risk families documented a five-time higher risk in non-carriers, compared with the general population, and reported a two-fold risk even in a prospective data subset. Several subsequent studies, though, did not confirm the presence of a significantly increased risk [3, 6, 14–17]. More recently, it has been tested if some non-carrier relatives from high-risk families might display a higher risk due to the effect of genetic modifiers, such as SNPs associated with an increased risk for BrCa either in the general population or in BRCA mutation carriers. However, this association was recorded only in *BRCA2* families [5].

In this study, we addressed for the first time whether the family mutation, in a mosaic state undetected by conventional screening methods, could underlie the development of BrCa in relatives testing negative. This could be explained by revertant somatic mosaicism, a phenomenon described at high frequency in hematological conditions, in particular primary immunodeficiencies and Fanconi’s anemia, and also observed in non-hematological disorders, such as epidermolysis bullosa, Duchenne muscular dystrophy and tyrosinemia [11]. Notably, revertant mosaicism predominantly involves tissues with high cell proliferation rates, as the hematopoietic system.

Following this hypothesis, in order to increase the chances of identifying the family mutation, we selected *BRCA1/2* families with non-carriers who were unexpectedly affected at an early age, most of them classified as type A phenocopies according to Smith et al [2]. By using techniques sensitive to low-level mosaicisms, we performed a mutation screening in blood and other available tissues. Of note, in six cases the analysis was carried out even on tumour samples, which ought to harbour the mutation if it were the primary causal factor. Our results confirmed the absence of the family mutation in all the analysed phenocopies, thus suggesting that a mutation reversion event is not expected to occur in such cases.

Interestingly, we observed that among phenocopies only three patients developed a high-grade BrCa, whereas most displayed low-intermediate hormone receptor positive BrCa (Table 1). This datum is noteworthy, since low-intermediate grade tumours are not frequently found in *BRCA1/2* families and are considered to be negative predictors of the BRCA mutation status [18]. In line with our results, this observation further points out that different pathomechanisms might underlie these cases. Moreover, in our cohort, four phenocopies also developed other tumours, which are not usually associated with the *BRCA1/2* phenotype. The presence of multiple primary tumours might be due to chance, or to the preceding use of genotoxic therapies, or might further indicate that a different type of susceptibility affect these individuals. The presence of a putative somatic mosaicism, in fact, is not expected to result in a phenotype more severe than usually observed in carriers of constitutional mutations. Notwithstanding, the inclusion of such cases in our study should not undermine our conclusions, as we avoided a selection biased towards specific phenotypes by including all the type A phenocopies in our series who developed BrCa below 50 years of age (with the exception of patient 4-e). Since BrCa is a very common disease in the general population, with most cases being sporadic, the age threshold was set in order to reduce the chances of including potential sporadic cases. This strategy was conceived based on the ratio of the age-specific BrCa rates in *BRCA1/2* carriers compared with the general population, which is estimated 20:1 in the age range 40–49 years, but drops down to 6:1 between 50–59 years and further decreases to 4:1 in the range 60–80 years [5]. Nevertheless, we can’t exclude that the selected phenocopies are actually a chance finding of naturally occurring sporadic cases in these families.

Although the BrCa risk for non-carrier relatives is not completely defined, the high phenocopy rate reported by many authors raises highly relevant issues. Primarily, phenocopies could be chosen as index cases and offered genetic testing before other affected relatives, who are mutation carriers. Nielsen and colleagues noted that in 3% of all families in their cohort, a phenocopy was tested as the first person in the family [6]. In addition, a study from the German Consortium for HBOC reported that among families tested negative at the first analysis, in whom another relative was offered *BRCA1/2* testing, about 5% (8/169) of second tests identified a mutation [4]. The incorrect choice of the first individual to be tested, inevitably leads to overlook pathogenic mutations, underestimate the disease penetrance in such families, and erroneously estimate the breast and ovarian cancer risks in all the relatives. To overcome this eventuality, we usually offer testing to a second family member if, without taking into account the first tested patient, the family still fulfils our selection criteria for the *BRCA1/2* analysis [12]. Notably though, if the patient from our family 8, who was the youngest affected individual, had been available for testing at the time of the first analysis, following our guidelines we would have missed the *de novo BRCA2* mutation identified in her sister.

Moreover, even if a *BRCA1/2* mutation is already identified within a family, the presence of early onset BrCa-affected non-carriers hampers accurate risk estimates for both mutation carriers and other negative family members. These considerations, on the whole, emphasize the importance of identifying the causal factors underlying BRCA phenocopies.

The *de novo* occurrence of the mutation identified in family 8 adds further complexity to both the risk assessment and genetic counseling, in particular when the family history is not informative. Reports of *de novo* mutations affecting the BRCA genes are extremely rare, to date only 11 cases have been described in the literature (five in *BRCA1* and six in *BRCA2*) [19–29]. Previous studies suggested that this event might be especially expected in early onset BrCa patients without family history of the disease. Therefore, due to the presence of premenopausal BrCa in both the proband and her sister, this finding was particularly surprising in our family 8.

Some authors suggested that the overall frequency of *de novo* events is estimated to be low, also due to a possible fertility advantage provided by BRCA mutations that survive selection pressure [30]. However, as usually probands’ parents are not routinely tested, it is important to underline that an undefined number of *de novo* mutations might have been overlooked. The correct identification of these cases would have major implications for the offer of genetic testing to the relatives of mutation carriers.

Finally, a recent study documented for the first time, through a next generation sequencing approach, a low-level constitutional mosaicism of a *de novo BRCA1* mutation, in a woman who developed a triple-negative BrCa at the age of 43 [28]. This remarkable finding not only demonstrated that mosaics for *BRCA1/2* mutations can occur, but it also highlighted that, in a subset of cases, a pathogenic mutation could remain undetected by a standard testing approach.

## Conclusions

This is the first study to investigate the presence of mosaicism for the family mutation in *BRCA1/2* phenocopies. Our results suggest that revertant somatic mosaicism is not likely to be a common phenomenon in *BRCA1/2* families.

As our families were selected for testing based on high-risk selection criteria, further studies are needed in order to discover other genetic factors potentially involved in the pathogenesis of most of these cases. The identification of causal factors in *BRCA1/2* phenocopies will have a significant impact on risk assessment and genetic counselling for both *BRCA1/2* mutation carriers and their relatives. Furthermore, the identification of a *de novo BRCA2* mutation in our cohort highlights that, although rare, this event should be taken into account in the evaluation of high-risk families.

## Supporting Information

S1 Fig Pedigrees of 14 families included in the study. Genetic analysis results (MUT, mutation carrier; WT, *wild-type*) are reported beneath tested individuals; neoplastic diseases and age at diagnosis in years (y) are reported beneath affected patients. NHL, non-Hodgkin lymphoma; CNS, central nervous system;?, cancer type and age unknown or uncertain; FH, family history.
(PDF)

S2 Fig Mass spectra of phenocopies (white arrows) and mutation carriers (black arrows) from families 9–15. Mutant (mut) and *wild type* (wt) alleles are discriminated by the difference in size. The analysis was carried out on DNA extracted from peripheral blood leukocytes in all the patients.
(PDF)

S1 Table Primers used for the matrix-assisted laser desorption ionization time-of-flight mass spectrometry (MALDI-TOF MS) and SNP genotyping assays. (DOCX)
