# Supplementary material for: Revertant mosaicism for family mutations is not observed in BRCA1/2 phenocopies
Source: PLoS One. 2017 Feb 15;12(2):e0171663. doi: 10.1371/journal.pone.0171663 (PMC5310879; doi:10.1371/journal.pone.0171663)
Supplement: S1 Fig — Genetic analysis results (MUT, mutation carrier; WT, wild-type) are reported beneath tested individuals; neoplastic diseases and age at diagnosis in years (y) are reported beneath affected patients. NHL, non-Hodgkin lymphoma; CNS, central nervous system;?, cancer type and age unknown or uncertain; FH, family history. (PDF) [file pone.0171663.s001.pdf]

Family 1 *BRCA1* c.1380dupA (p.Phe461Ilefs*19)

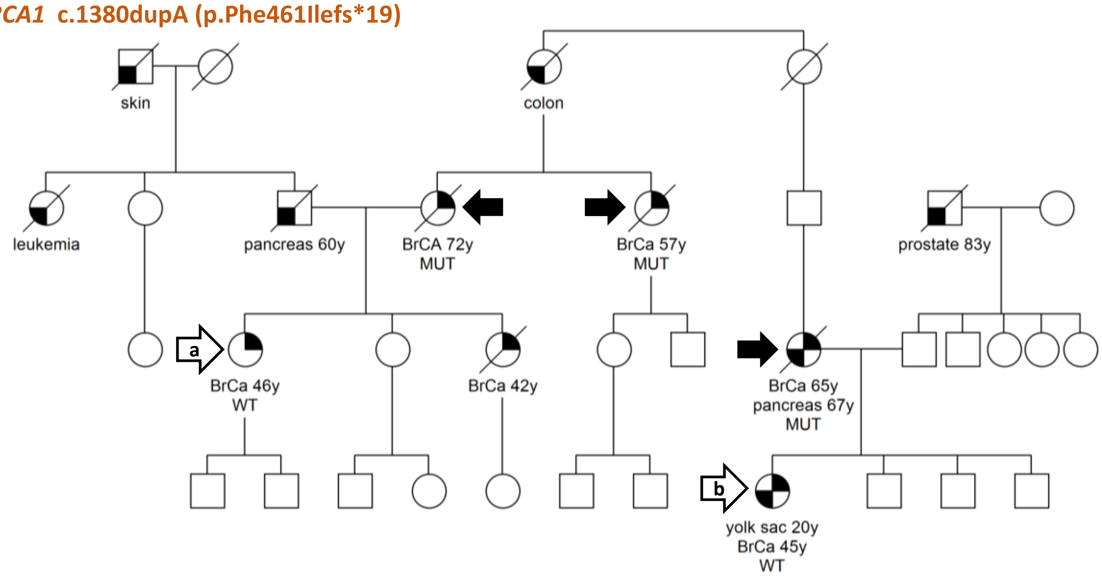

Family 2 *BRCA2* c.3109C>T (p.Gln1037*)

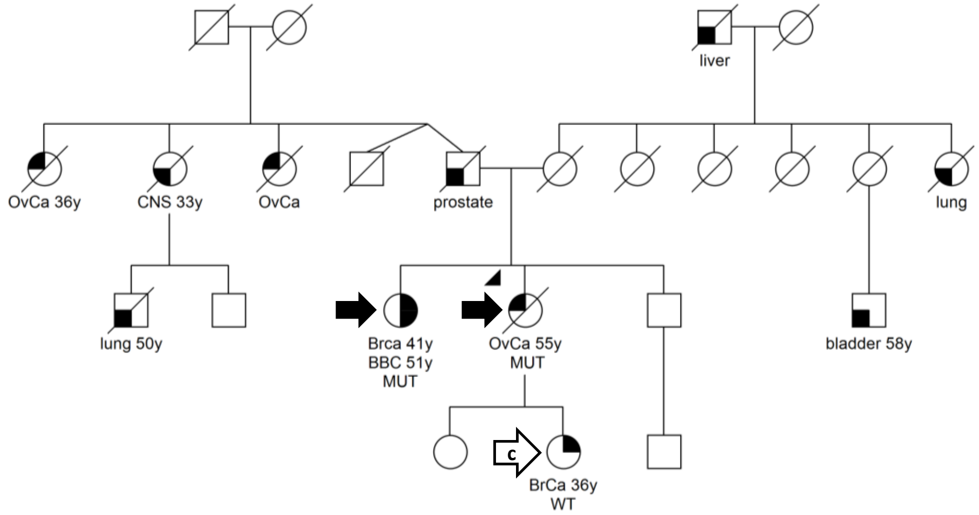

Family 3 *BRCA1* c.190T>C (p.Cys64Arg)

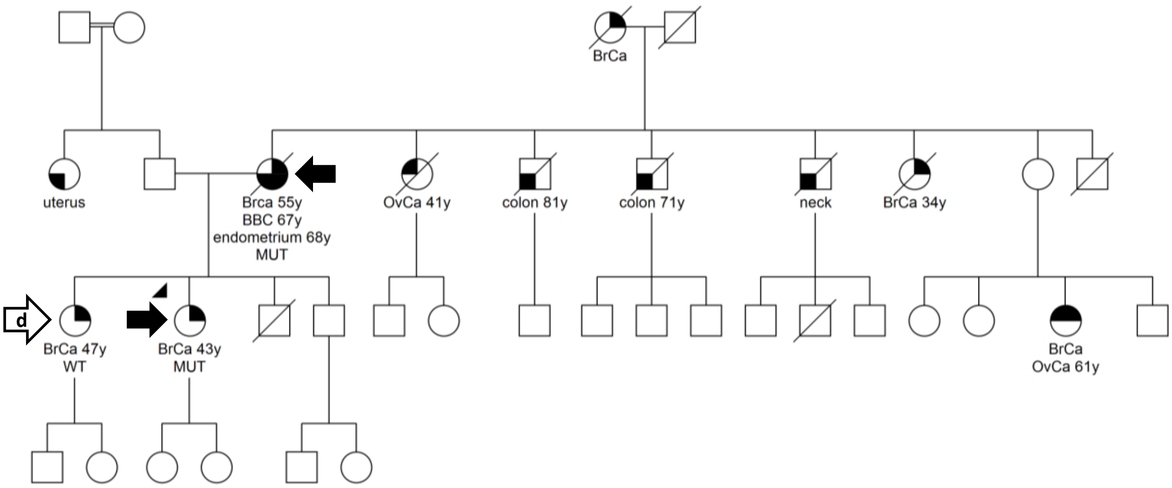

Family 4 *BRCA1* c.5266dupC (p.Gln1756Profs*74)

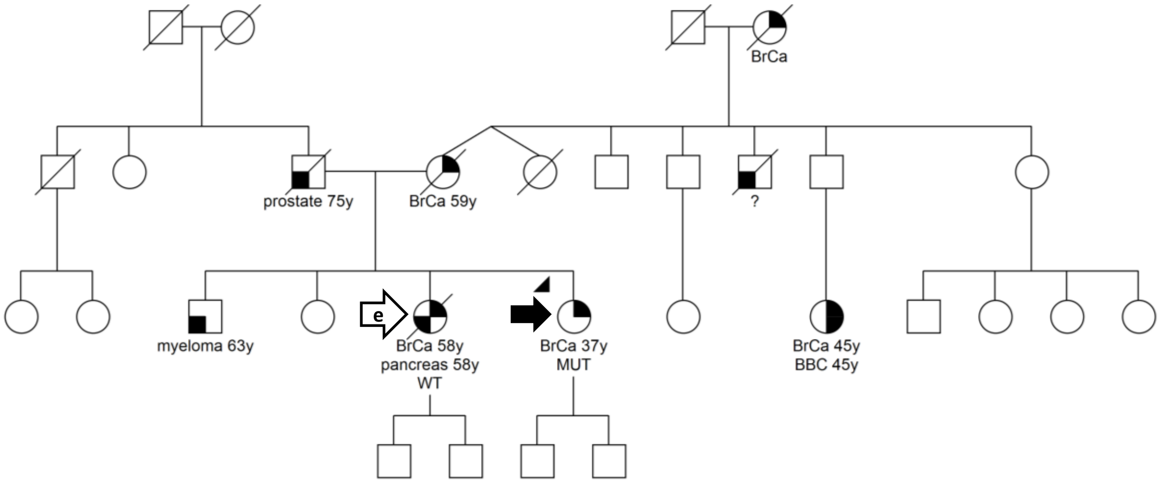

Family 5 *BRCA1* c.1088delA (p.Asn363Ilefs*11)

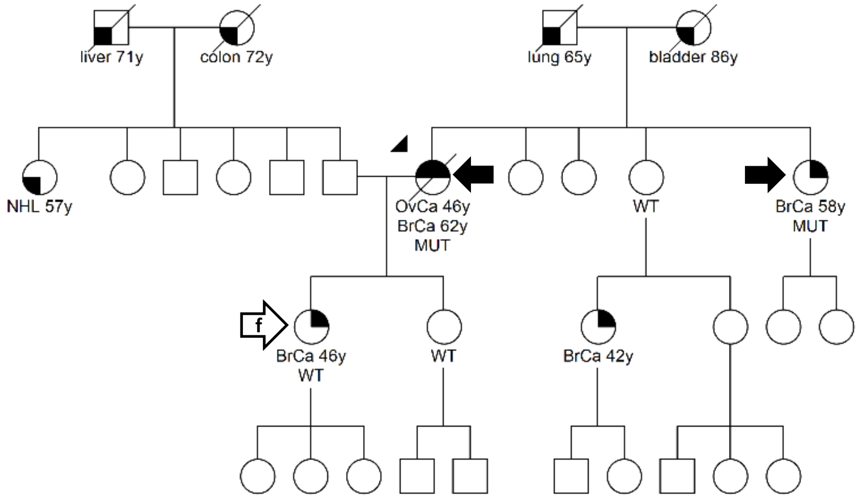

Family 6 *BRCA1* c.2157_2160delAGAA (p.Lys719Asnfs*16)

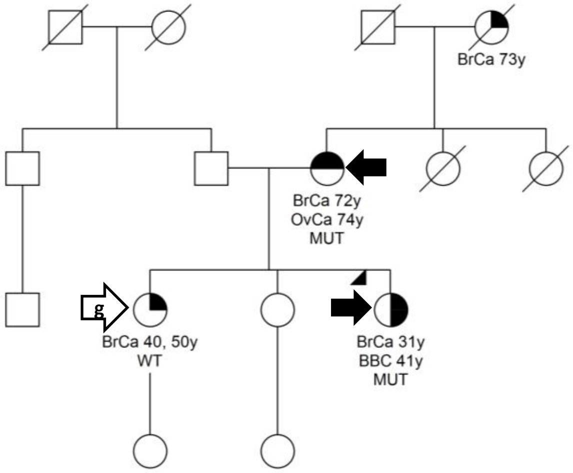

Family 7 *BRCA1* c.190T>C (p.Cys64Arg)

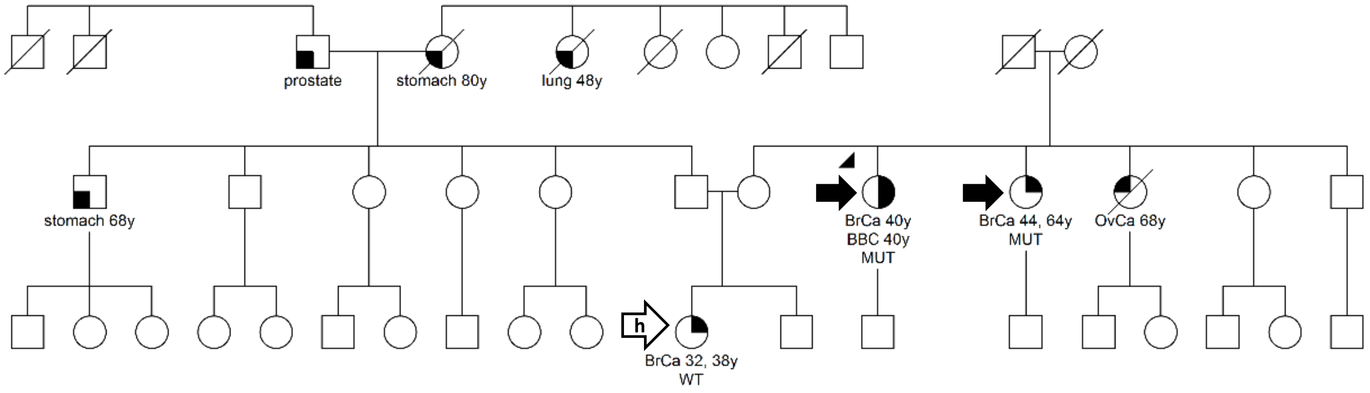

Family 9 *BRCA1* c.144delG (p.Met48Ilefs*2)

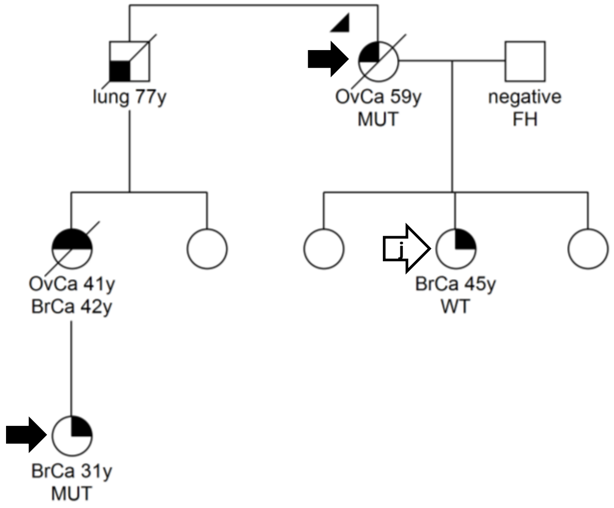

Family 10 *BRCA2* c.8487+1G>A

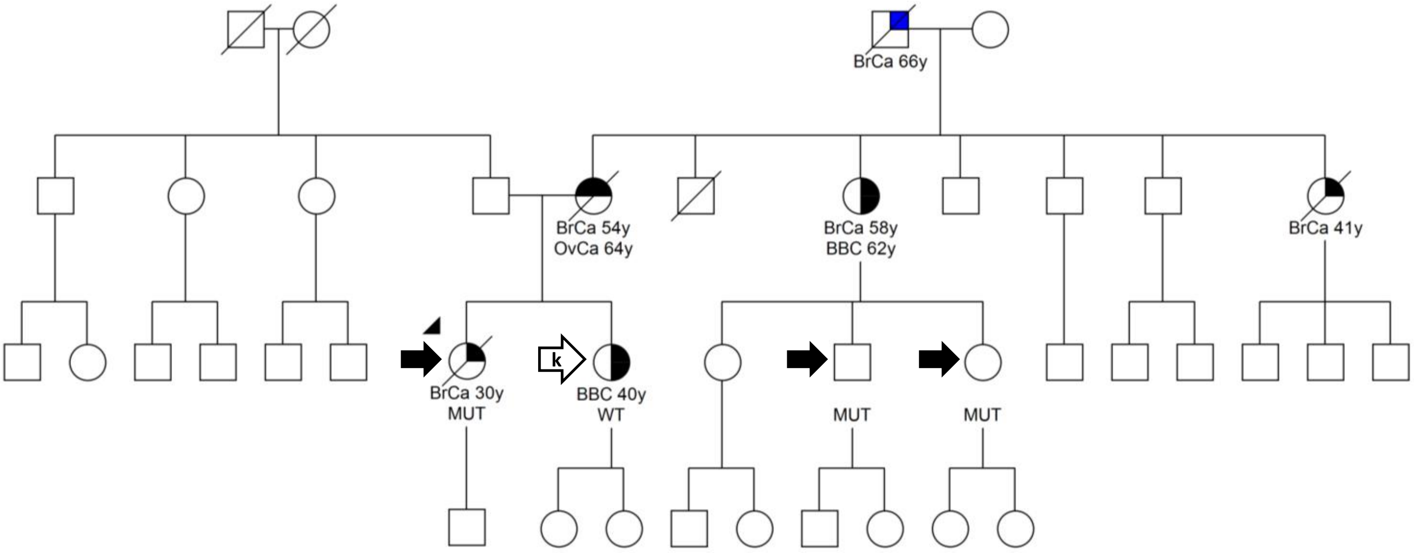

Family 11 *BRCA2* c.7806-2A>G

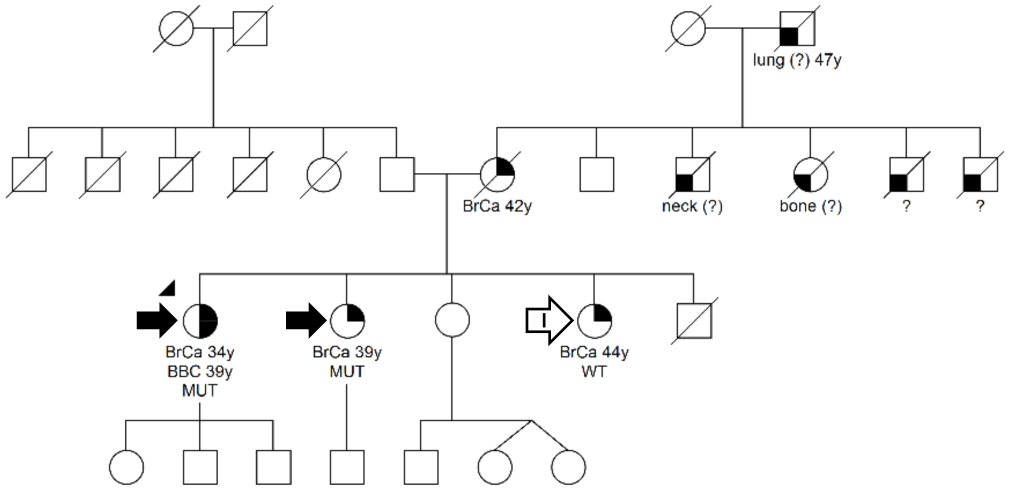

Family 12 *BRCA2* c.8754+4A>G (p.G2919Vfs*4)

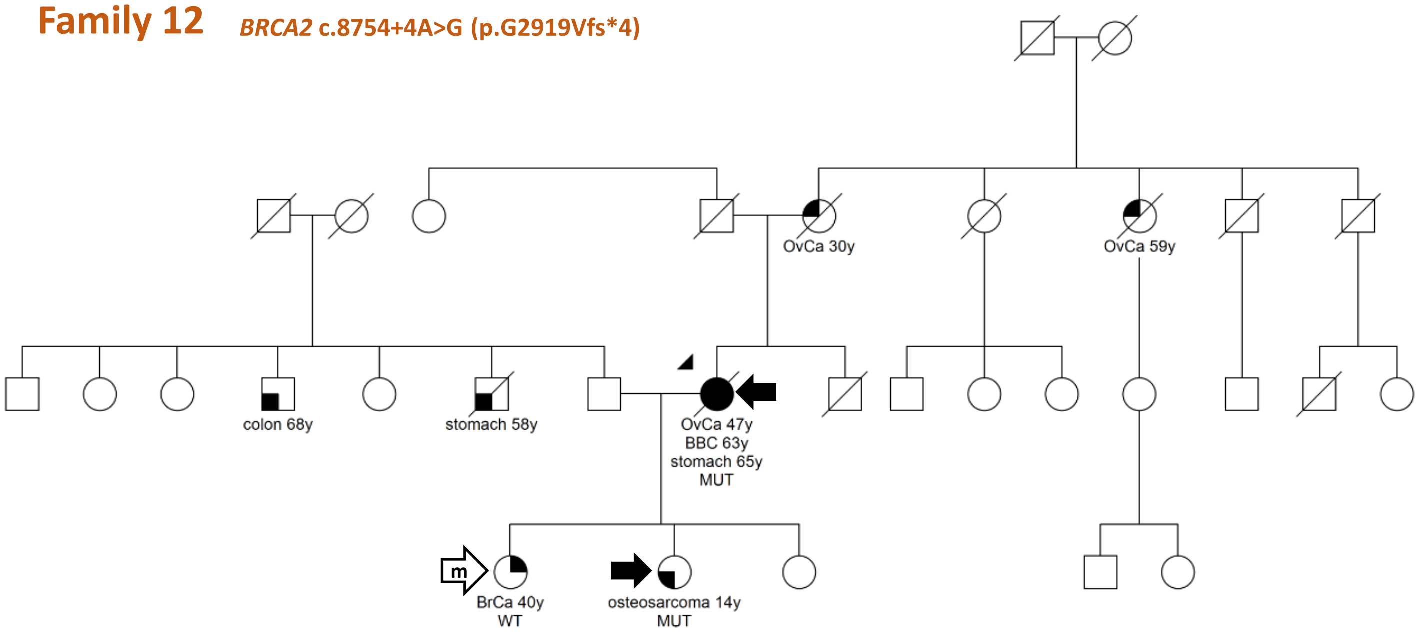

Family 13 *BRCA1* c.981_982delAT (p.Cys328*)

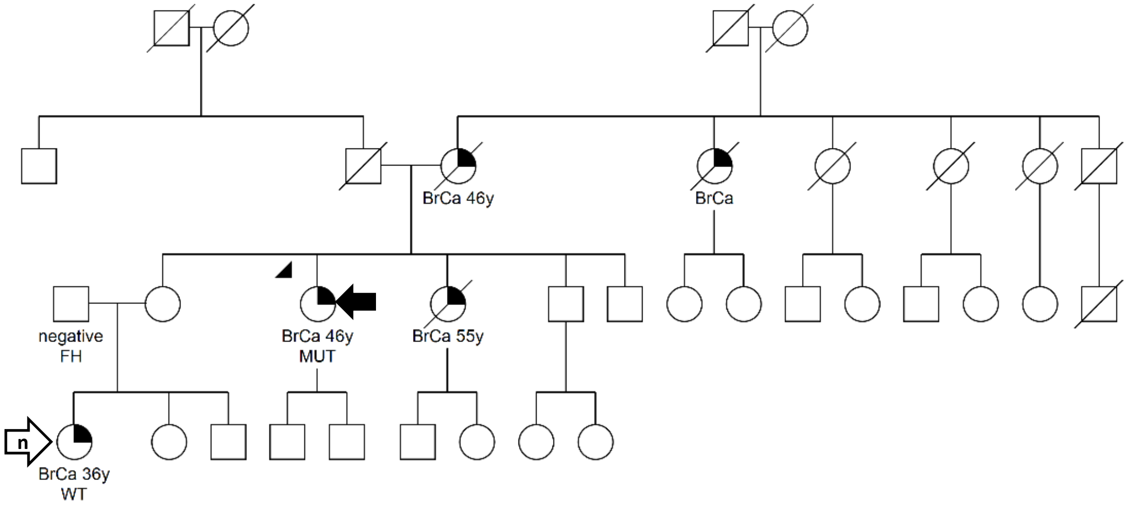

Family 14 *BRCA1* c.798_799delTT (p.Ser267Lysfs*19)

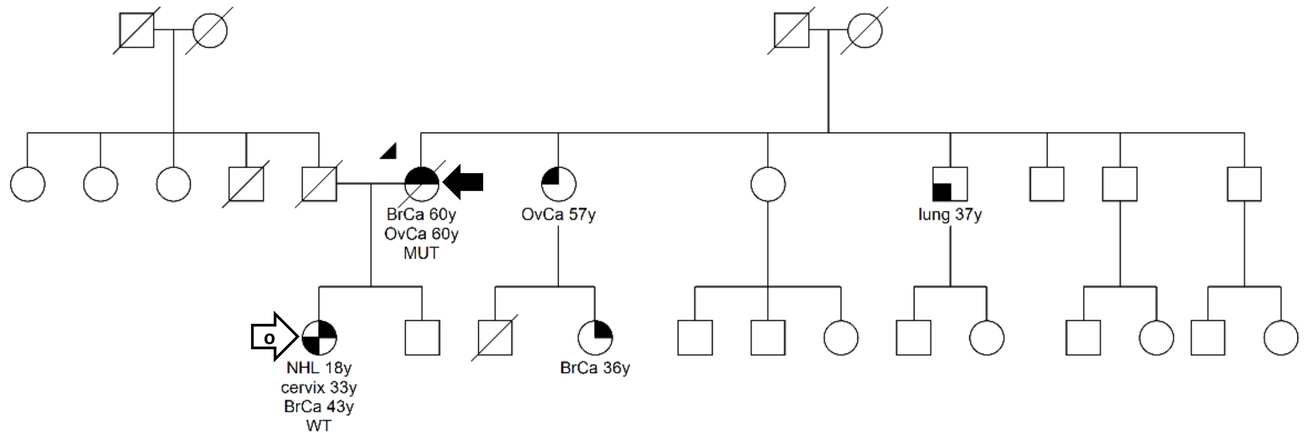

Family 15 *BRCA1* c.190T>C (p.Cys64Arg)

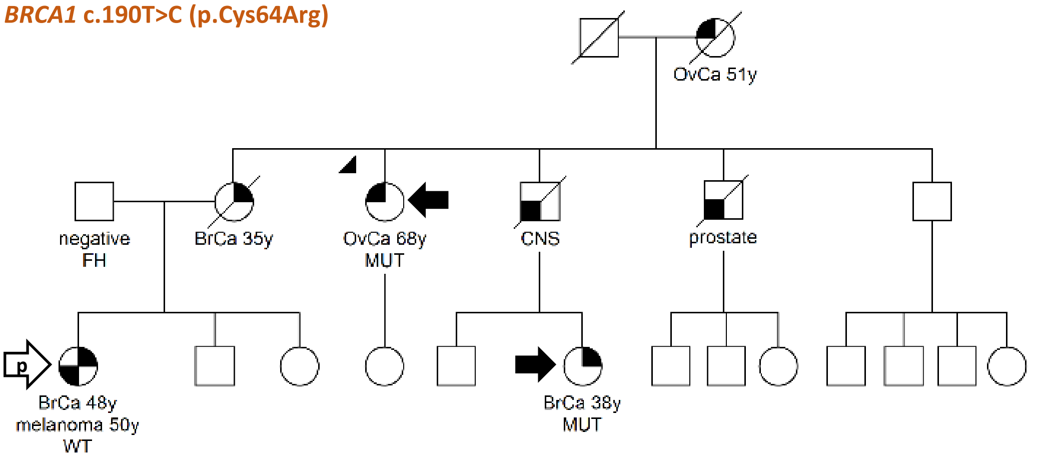

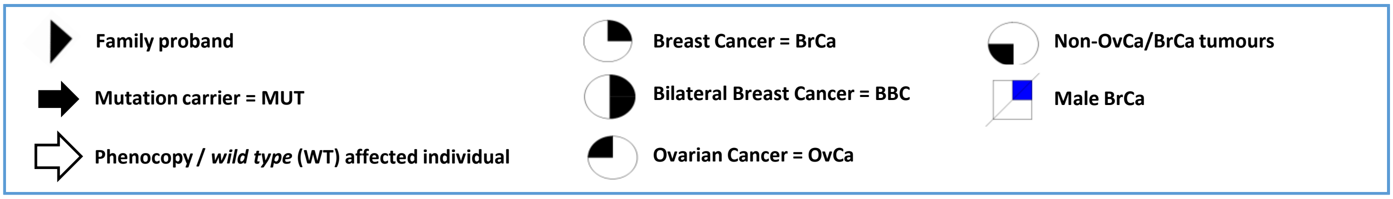

Supplementary Figure 1. Pedigrees of 14 families included in the study (family 8 is shown in Figure 2). Genetic analysis results (MUT, mutation carrier; WT, *wild-type*) are reported beneath tested individuals; neoplastic diseases and age at diagnosis in years (y) are reported beneath affected patients. NHL, non-Hodgkin lymphoma; CNS, central nervous system; ?, cancer type and age unknown or uncertain; FH, family history.
