# Supplementary material for: Revertant mosaicism for family mutations is not observed in BRCA1/2 phenocopies
Source: PLoS One. 2017 Feb 15;12(2):e0171663. doi: 10.1371/journal.pone.0171663 (PMC5310879; doi:10.1371/journal.pone.0171663)
Supplement: S2 Fig — Mutant (mut) and wild type (wt) alleles are discriminated by the difference in size. The analysis was carried out on DNA extracted from peripheral blood leukocytes in all the patients. (PDF) [file pone.0171663.s002.pdf]

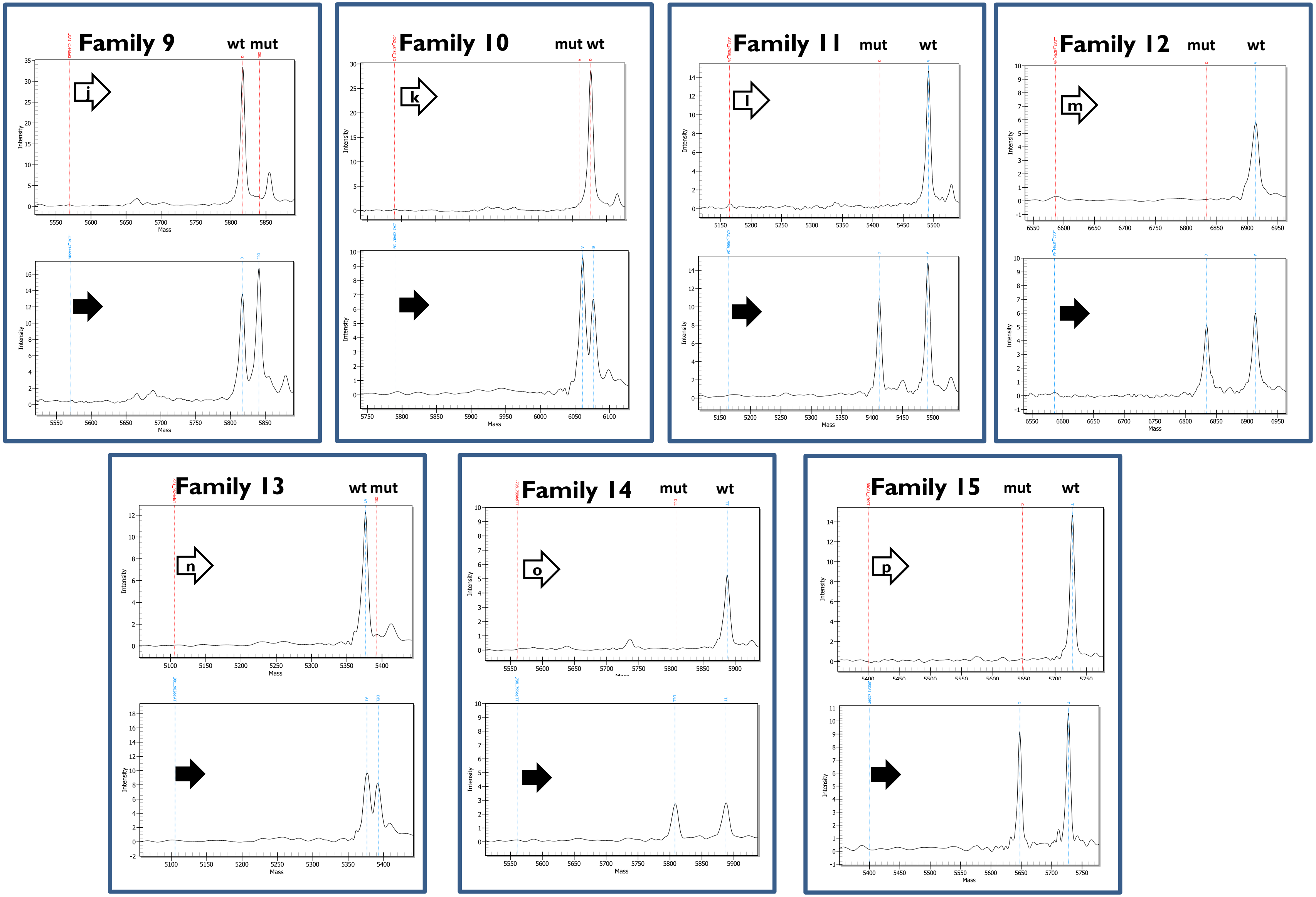

Supplementary Figure 2. Mass spectra of phenocopies (white arrows) and mutation carriers (black arrows) from families 9-15. Mutant (mut) and *wild type* (wt) alleles are discriminated by the difference in size. The analysis was carried out on DNA extracted from peripheral blood leukocytes in all the patients.
