# Supplementary material for: Revertant mosaicism for family mutations is not observed in BRCA1/2 phenocopies
Source: PLoS One. 2017 Feb 15;12(2):e0171663. doi: 10.1371/journal.pone.0171663 (PMC5310879; doi:10.1371/journal.pone.0171663)
Supplement: S1 Table — (DOCX) [file pone.0171663.s003.docx]

| **MALDI-TOF MASS SPECTROMETRY PRIMERS TABLE** |
| --- |

| GENE | MUTATION | PRIMERS | AMPLICON (bp) | |
| --- | --- | --- | --- | --- |
| BRCA1 | C.1499insA | Fw_ACGTTGGATGAAGAGTTCACTCCAAATCAG | | 98 |
|  |  | Rev_ACGTTGGATGGGCTTGCCTTCTTCCGATAG | |  |
|  |  | Seq_AGAGAGTAATATTGAAGACAAAATA | |  |
|  | c.309 T>C | Fw_ACGTTGGATGAGAAGAAAGGGCCTTCACAG | | 121 |
|  |  | Rev_ACGTTGGATGTTTCCTACTGTGGTTGCTTC | |  |
|  |  | Seq_CCTTCACAGTGTCCTTTA | |  |
|  | c.5382insC | Fw_ACGTTGGATGTTTGTCAACTTGAGGGAGGG | | 95 |
|  |  | Rev_ACGTTGGATGAAACCACCAAGGTCCAAAGC | |  |
|  |  | Seq_TACCTTTCTGTCCTGGG | |  |
|  | c.1207delA | Fw_ACGTTGGATGATCTGAATGCTGATCCCCTG | | 115 |
|  |  | Rev_ACGTTGGATGCCAAGGAACATCTTCAGTATC | |  |
|  |  | Seq_ACTGCCATGCTCAGAGA | |  |
|  | c.2157_2160delAGAA | Fw_ACGTTGGATGTCTTCTCTTGGAAGGCTAGG | | 94 |
|  |  | Rev_ACGTTGGATGCCTGGTTCTTTTACTAAGTG | |  |
|  |  | Seq_TTCAAATACCAGTGAACTTAA | |  |
|  | c.190 T>C | Fw_ACGTTGGATGAGGCTCCTTTTGGTTATAC | | 86 |
|  |  | Rev_ACGTTGGATGTCTCAACCAGAAGAAAGGGC | |  |
|  |  | Seq_CCTTCACAGTGTCCTTTA | |  |
| BRCA2 | c.3109 C>T | Fw_ACGTTGGATGCAACACAAGCTAAACTAGTA | | 112 |
|  |  | Rev_ACGTTGGATGGGAAATCAAGCTCTCTGAAC | |  |
|  |  | Seq_CAAGCTAAACTAGTAGGATATT | |  |
|  | c.771_775delTCAAA | Fw_ACGTTGGATGTTATCGCTTCTGTGACAGAC | | 87 |
|  |  | Rev_ACGTTGGATGGAGGACTTACCATGACTTGC | |  |
|  |  | Seq_CAGACAGTGAAAACACAAA | |  |

| **TAQMAN SNP GENOTYPING ASSAY** | | | | | |
| --- | --- | --- | --- | --- | --- |
| **MUTATION** | **PRIMERS** | **Tm (°C)** | **CG**  **(%)** | **Length (bp)** | **Fluorescent Dye** |
| *BRCA2* | Fw_GATAGATTTATCGCTTCTGTGACAGACA | 60 | 39 | 28 |  |
| c.771_775delTCAAA | Rev_ACCTGTAGTTCAACTAAACAGAGGACTT | 57,4 | 39 | 28 |  |
|  | Wild-type allele AAACACAAA**TCAAA**GAGAAG | 67 | 30 | 20 | FAM |
|  | Mutated allele TGAAAACACAAAGAGAAG | 66 | 33 | 18 | VIC |

**Supplementary Table 1**. Primers used for the matrix-assisted laser desorption ionization time-of-flight mass spectrometry (MALDI-TOF MS) and SNP Genotyping assays.
